# Building a country-wide Fistula Treatment Network in Kenya: results from the first six years (2014-2020)

**DOI:** 10.1186/s12913-021-07351-x

**Published:** 2022-03-01

**Authors:** Lindsey Pollaczek, Alison M. El Ayadi, Habiba C. Mohamed

**Affiliations:** 1 Fistula Foundation, 95126 San Jose, CA USA; 2 Department of Obstetrics, Gynecology and Reproductive Sciences, University of California San Francisco, San Francisco, CA USA; 3 Fistula Foundation, Mumias, Kenya

**Keywords:** Obstetric fistula, Vesico-vaginal fistula, Female genital fistula, Obstructed labor, Maternal morbidity, Fistula surgeons, Community health volunteers, Holistic care, Multicomponent intervention, Kenya, Fistula Foundation, Fistula Treatment Network, Network of Care

## Abstract

It is estimated that one million women worldwide live with untreated fistula, a devastating injury primarily caused by prolonged obstructed labor when women do not have access to timely emergency obstetric care. Women with fistula are incontinent of urine and/or feces and often suffer severe social and psychological consequences, such as profound stigma and depression. Obstetric fistula affects economically vulnerable women and garners little attention on the global health stage. Exact figures on fistula incidence and prevalence are not known. In Kenya, results from a 2014 population-based survey suggest that 1% of reproductive-aged women have experienced fistula-like symptoms.

In collaboration with key stakeholders, Fistula Foundation launched the Fistula Treatment Network (initially known as Action on Fistula) in 2014 to increase access to timely, quality fistula treatment and comprehensive post-operative care for women with fistula in Kenya. The integrated model built linkages between the community and the health system to support women through all parts of their treatment journey and to build capacity of healthcare providers and community leaders who care for these women. Fistula Foundation and its donors provided the program’s funding. Seed funding, representing about 30% of the program budget, was provided by Astellas Pharma EMEA.

Over the six-year period from 2014 to 2020, the network supported 6,223 surgeries at seven hospitals, established a fistula training center, trained eleven surgeons and 424 Community Health Volunteers, conducted extensive community outreach, and contributed to the National Strategic Framework to End Female Genital Fistula. At 12 months post fistula repair, 96% of women in a community setting reported that they were not experiencing any incontinence and the proportion of women reporting normal functioning increased from 18% at baseline to 85% at twelve-months.

The Fistula Treatment Network facilitated collaboration across hospital and community actors to enhance long-term outcomes for women living with fistula. This model improved awareness and reduced stigma, increased access to surgery, strengthened the fistula workforce, and facilitated post-operative follow-up and reintegration support for women. This integrated approach is an effective and replicable model for building capacity to deliver comprehensive fistula care services in other countries where the burden of fistula is high.

**Supplementary Information:**

The online version contains supplementary material available at 10.1186/s12913-021-07351-x.

## Background

Obstetric fistula is a devastating childbirth injury primarily caused by prolonged obstructed labor combined with lack of access to emergency obstetric care, specifically a timely cesarean section. Despite being both preventable and largely treatable, women of all ages can develop obstetric fistula in the absence of appropriate care. Following the Three Delays Model commonly used to describe circumstances contributing to maternal mortality - delay in the decision to seek care; delay in arrival at a health facility; and delay in the provision of adequate care—fistula formation can occur when women experience delays in accessing critical obstetric care due to factors of distance, cost, and quality [1, 2].

Obstetric fistula is a chronic disability that has severe physical, psychological, social, and economic consequences. Women with obstetric fistula experience constant leaking of urine and/or feces through the vagina and in addition can experience stillbirth, excoriation of the skin, amenorrhea, vaginal stenosis, infertility, orthopedic injury, and neurologic disorders [3]. Due to the strong smell associated with incontinence and misperceptions about the cause of the fistula, many women experience severe stigma and isolation and can suffer from depression and suicide ideation. Many women with fistula are outcast from their husbands and families, and living with the condition makes it even harder for them to earn income, further driving them into poverty.

Obtaining accurate prevalence or incidence data for fistula is notoriously difficult. Women with fistula tend to be poor, live in hard-to-reach rural areas, and are very often socially isolated [4–6]. Furthermore, fistula is a relatively rare morbidity for which data are frequently available only from clinical settings; accurate estimation of the number of women living with fistula in the community via population-representative surveys is challenged by the low prevalence combined with the need for clinical diagnosis [7, 8]. Current available estimates on the number of women globally living with fistula range from one million to as many as two million [9]. Incidence is estimated at 50,000-100,000 new cases per year [10].

Reconstructive surgery is the primary form of treatment for women with obstetric fistula, and it has been shown to significantly improve a woman’s physical and psychological health [11–13]. According to Landry et al., women from five countries in Africa and Asia reported significant improvements to their quality of life just three months post-surgery, including their ability to eat with others, work, and attend social gatherings [4]. However, many women often wait a long time for fistula surgery due to lack of awareness or knowledge about the condition, limited or inaccessible transportation to a surgical facility where treatment is available, and lack of adequately trained surgeons, full time nursing care, or equipped facilities [2]. This delay in treatment access has been termed “the fourth delay” [14].

The international response to address the challenge of obstetric fistula was spearheaded by the United Nations Population Fund (UNFPA) and partners in 2003 with the launch of the global Campaign to End Obstetric Fistula. The campaign has focused on fistula prevention, treatment, rehabilitation, and reintegration for women with fistula and is currently active in over 55 countries [15].

### Efforts to Address Fistula in Kenya

Kenya was an early participant in the global Campaign to End Fistula in 2004. Since then, the Kenyan Ministry of Health (MOH) and Division of Family Health and Reproductive and Maternal Health Services Unit have instituted many strategies and policies aimed at reducing maternal mortality and morbidity, including the removal of all delivery fees at public hospitals in 2013. Such policies should have decreased the incidence of fistula and other maternal morbidities but did not expressly focus on increasing access to fistula treatment. The Kenya Health Sector Strategic and Investment Plan (2018-2023): Transforming Health Systems: Achieving Universal Health Coverage by 2022 sets targets for improving coverage of essential obstetric services but does not specifically outline a plan for addressing obstetric fistula [16]. Since the launch of the Campaign to End Fistula, multiple non-governmental organizations (NGOs) have joined the MOH and UNFPA to support fistula treatment activities, including AMREF Health Africa, Direct Relief, Freedom from Fistula Foundation, Flying Doctors Society of Africa, MUMCOP, One By One, and Women and Development against Distress in Africa (WADADIA) [17–20].

Needs assessments jointly conducted by the Kenyan MOH and UNFPA in 2004 found the actual fistula burden in the country difficult to quantify; however, they estimated that 3,000 new fistula cases occurred each year, with approximately one to two fistulae for every 1,000 deliveries [21] The latest Kenyan Demographic and Health Survey (2014) reported a 1% lifetime prevalence of women aged 15-49 self-reporting with fistula-like symptoms [22, 23]. Other indicators of maternal health suggest ongoing challenges: Kenya’s maternal mortality ratio remains high at 362 maternal deaths per 100,000 live births, and significant disparities exist between urban and rural populations in the proportion of women delivering in a health facility or with a skilled provider (50% vs. 82%, for urban and rural inhabitants, respectively) [22].

It is difficult to obtain reliable data on the number of fistula surgeries conducted annually in Kenya. Stakeholders track surgeries supported through their organizations but reporting to the Ministry of Health is inconsistent. The Global Obstetric Fistula Hub (formerly Global Fistula Map), a collaborative effort of UNFPA, Fistula Foundation, and Direct Relief that aimed to improve visibility of provision of fistula surgery, shows an average of 822 fistula surgeries completed annually between 2010 and 2013. However, this data is self-reported and may be incomplete [24].

Prior to 2014, routine fistula surgery was rare and was primarily done in three hospitals countrywide—two of which were based in Nairobi [25]. Long distances to Nairobi, cost of transport, and fear and anxiety in traveling to an unknown urban center resulted in many women being unable to access these routine services [26, 27]. Most fistula surgery was done through fistula repair “camps”—short-term mobilization of women with suspected fistula, screening, and repair for those affected at provincial or district hospitals under guidance from visiting expert surgeons. Camps were held roughly once a year—or when funding from a donor partner was available—and focused on particular regions. The temporal nature of these camp services meant that not every woman living with fistula was reached or able to receive fistula treatment in a timely manner [25].

Limited availability of trained fistula surgeons has been a chronic issue in addressing the need for fistula care in Kenya. Prior to 2014, training of fistula surgeons was done primarily through the fistula repair camps. Health providers reported that these venues were useful for mentorship of surgeons; however, the intention of having the surgeon continue to provide routine surgery after the end of the camp was seldom achieved. In addition to the limited ongoing mentorship available after the camp ended, lack of available operating theater space at busy public hospitals, limited bed space for post-operative care, and limitations in availability of essential supplies and equipment made providing routine services very difficult [25].

Low awareness of fistula, high rates of stigma, and misperceptions about the condition further hindered women’s ability to access treatment [25–27]. Civil society organizations involved in fistula programming worked to reach communities with information about fistula causes, prevention, and treatment availability. Mass media like radio was commonly used as a key mobilization tool prior to fistula repair camps. One regional program in western Kenya, *Let’s End Fistula*, a collaboration between three organizations, focused on training fistula survivors to be community ambassadors to help build trust in the community, improve referrals to the treatment center, and provide support for women when they returned to their communities post discharge [28]. Reintegration and rehabilitation of women after fistula surgery are broadly recognized as an important aspect of fistula care, albeit one that has not received much attention in Kenya, or in the broader global community [29, 30]. Even after successful closure of a fistula, many women may still suffer social stigma, economic hardship, and possible fistula reoccurrence [31].

Given these demonstrated needs, Fistula Foundation developed and implemented a country-wide, integrated framework to support routine treatment and reintegration services for women with obstetric fistula. In the current paper, we report on the Fistula Treatment Network (FTN) model in Kenya and achievements during the first six years of the project, from May 2014 to April 2020.

## Methods

### Conceptualizing the Fistula Treatment Network (FTN)

The vision of FTN was an interconnected model to increase access to timely, quality fistula treatment and comprehensive post-operative care for women with fistula. FTN sought to build linkages between the community and the health system to facilitate women through all stages of their healing journey, and to build capacity of healthcare providers and community leaders who care for these women. FTN trained surgeons and nurses and launched fistula services at a network of hospitals that provided free fistula surgery on a routine basis. To facilitate movement of women between the community and the hospital setting, FTN invested in community organizations that improved patient identification, referral, and follow-up after discharge to promote healing and reintegration. This was a unique model in fistula care that sought to deepen collaboration between hospital and community partners to optimize the best outcomes for women. It is consistent with a “Network of Care” framework in its approach to connecting service delivery partners to promote a structure that prioritizes client-centered care and enables providers across all levels of care to work in teams and share responsibility for health outcomes [32].

The FTN vision was conceived in 2013 by Fistula Foundation’s CEO, Kate Grant, and was launched in 2014 by Fistula Foundation’s Vice President of Programs, Lindsey Pollaczek. Fistula Foundation provided grant funding to cover the full cost of the FTN, including fistula care and treatment, surgeon and health provider training, and community outreach and reintegration activities.

**Fig. 1:**
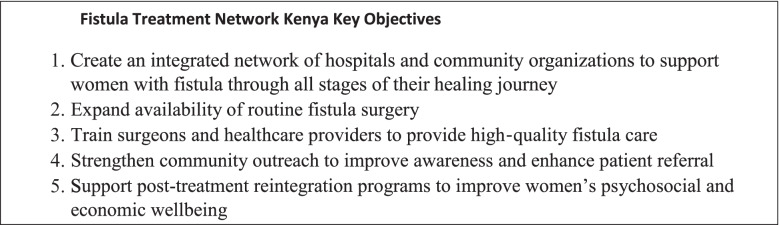
Fistula Treatment Network Kenya Key Objectives

**Fig. 2:**
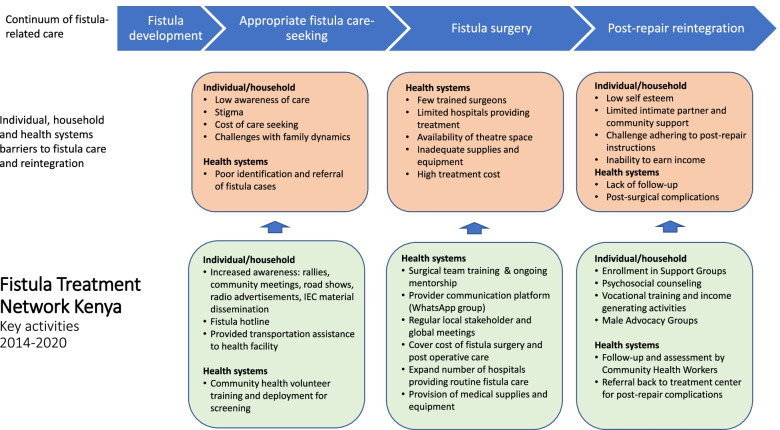
Conceptual framework of gaps in fistula continuum of care and Fistula Treatment Network Kenya key activities, 2014-2020

 In collaboration with key stakeholders in Kenya, FTN developed the following objectives: create an integrated network of hospital and community service providers to support women with fistula through all stages of their healing journey; expand availability of routine fistula surgery; train surgeons and healthcare providers in high quality fistula care; strengthen community outreach to improve awareness and enhance patient referral; and support post-treatment reintegration programs to improve women’s psychosocial and economic wellbeing (Fig. 1). These objectives were selected based on their role in addressing key barriers to access to fistula care and reintegration in Kenya at the individual, household, and health systems levels. In Fig. 2 we depict the main barriers that limited fistula-care seeking, fistula surgery, and post-repair reintegration, and the specific activities implemented by FTN to overcome these barriers.

1. Create an integrated network of hospitals and community organizations to support women with fistula through all stages of their healing journey

FTN conducted stakeholder meetings with the MOH, fistula surgeons, hospital administrators, and Kenyan NGOs and community-based organizations (CBOs) working in fistula programming to assess the current landscape, gaps, and opportunities for growth. When the program was launched in 2014, FTN partners included the MOH, four hospitals, and three outreach organizations. Throughout program implementation, FTN strategically expanded this stakeholder network to meet the needs of women affected by fistula throughout the country by incorporating additional hospitals, outreach organizations, and counties of coverage in order to create the linkages needed between community and health systems actors. Further information on linkages across programs are described below by activity type.

2. Expand availability of routine fistula surgery

In response to FTN’s landscape assessment of gaps in fistula treatment and opportunities for growth, we selected regional hubs for routine fistula service expansion to form the network based on perceived fistula burden. Due to poor community-based prevalence data, we used proxy measures of percentage of facility-based deliveries and percentage of births attended by a skilled provider, as well as personal communication with surgeons with experience working in specific regions. Priority was given to locations where (i) women were not presenting for care due to low awareness or lack of transport funds; (ii) we could leverage existing assets, such as a pre-trained fistula surgeon; (iii) routine services were not available or limited due to high user fees for fistula surgery or limited surgical equipment, supplies, theater time, and ward space. We sought locations where hospital administrators and county officials demonstrated strong support to invest in a routine service over a camp-based approach. Understanding the importance of robust community mobilization, we considered regions where we could build partnerships with existing, reliable CBOs or NGOs working in reproductive health services that would be able to create strong linkages between the communities and the treatment centers.

3. Train surgeons and healthcare providers to provide high-quality fistula care

FTN worked in partnership with the International Federation of Gynecology and Obstetrics (FIGO) to provide targeted training for fistula surgery in the country. In 2014, FIGO representatives accredited the FIGO training center at Gynocare Women’s and Fistula Hospital in Eldoret. That same year, four Kenyan surgeons attended a Training of Trainers event in Tanzania, to be qualified as surgeon trainers using the FIGO and Partners *Global Competency Based Fistula Training Manual*, [33] the training curriculum used by FTN.

After certification of the trainers, a Review Committee was established to review prospective applicants for acceptance into the training program using the FIGO criteria. The criteria included whether the applicant had practiced surgery for a minimum of three years, whether the applicant was supported by their institution and county government to attend a six-week residential training, and whether the applicant was likely to immediately and on a long-term basis put their newly gained skills to use upon returning to their home facility.

4. Strengthen community outreach to improve awareness and enhance patient referral

A cornerstone of FTN was the development of a robust outreach program to strengthen the linkage between the community and the hospital setting. In 2014, FTN partnered with the Kenyan NGO Women and Development Against Distress in Africa (WADADIA), an organization with experience in fistula programming at the community level. FTN provided a grant to WADADIA to enable the organization to expand into more counties of western Kenya. Additional community organizations were recruited over the course of the six years to join FTN and received training and grants to implement outreach in their respective coverage areas. These organizations were selected based on their track record of strong community engagement, their focus on sexual and reproductive health and gender equality issues, and their geographic catchment areas—to ensure they were linked to a referral center where treatment was available.

5. Support post-treatment reintegration programs to improve women’s psychosocial and economic wellbeing

After hospital discharge, some women have ongoing psychological, social, and economic needs, and benefit from support to reintegrate with their communities and families. The goals of the FTN reintegration program were to enhance fistula survivors’ quality of life through holistic recovery and to prevent fistula recurrence. The three key pillars of the FTN reintegration model were psychosocial support, economic empowerment, and reproductive health services. The reintegration component of FTN was adopted following lessons learned from the first phase of the program (2014-2017). Working its community outreach partners, who had already developed a high level of trust within their communities, FTN began to support key reintegration activities in May 2017. FTN’s reintegration programs included follow-up visits, community support groups, income-generating activities, vocational training, educational support, and the creation of male advocacy groups.

### Consent to participate

At the community level, women who were identified through FTN partners were counseled and prepared for what to expect during their referral, treatment, and reintegration process. Family members were involved whenever possible so that both the woman and the family understood what was involved in fistula treatment as well as in the post-operative reintegration period. All women provided written consent (thumb print for non-literate patients) for referral to the treatment center and further evaluation. Following diagnosis at the hospital and pre-operative counseling, another signed consent was collected by the hospital staff prior to the surgical intervention.

### Monitoring and Evaluation

Program primarily collected from two sources: the hospitals providing the fistula surgery and the community outreach organizations focused on patient identification, referral, and reintegration post-repair.

At the hospitals, surgical data was directly entered onto a standardized form appended to the hospital medical record. At the end of each month, the resident fistula surgeon and program manager from each hospital entered the data onto a standardized data collection instrument in Microsoft Excel and submitted it to the Fistula Foundation Kenya Office. Data quality was assessed by the Fistula Foundation Monitoring and Evaluation (M&E) Officer, and any discrepancies or incomplete data was clarified immediately, with payment to the hospital contingent on accurate and complete reporting. Fistula Foundation staff made quarterly on-site visits to review physical patient files to ensure completeness of patient records, including signed consent forms, and consistency with data submitted on the data collection instrument. Foundation staff called a random sample of 15-25% of patients treated at each facility each month (a minimum of 2-5 patients) to validate their treatment and outcome records. In addition, the Fistula Foundation Medical Advisor, an experienced fistula surgeon, reviewed the data and the progress at the facility and followed up on any clinical questions or opportunities for mentorship.

At the community level, data was managed by the five Kenyan NGOs/CBOs responsible for implementation of the community outreach and reintegration activities. This data included records on patient location, symptoms of fistula, outcomes of verbal screening, referral, and post-repair outcomes related to continence status and psychosocial wellbeing. Data was collected in the field by Community Health Volunteers and underwent multiple levels of review before transmission to the M&E Officer at each organization. At this point data was validated and entered into a standardized Excel database used across all FTN outreach teams and submitted to the M&E Officer on a quarterly basis. The community outreach teams completed a referral form for each woman sent to the treatment center. At hospital discharge, clinical staff added her clinical data and follow-up treatment plan to this form for the community-based team to facilitate the woman’s community-based follow-up needs (for example, in case she required a follow-up visit for surgery or other care).

 Review meetings for outreach teams and treatment centers were conducted routinely to discuss data and other programmatic issues, including an Annual Stakeholder Meeting to improve coordination and communication between the teams and enhance holistic patient care and treatment. Finally, annual audits of hospital and outreach teams were conducted by an independent auditor to assess the veracity of the financial and programmatic reports.

### Measures

Hospital data captured basic patient characteristics including county of residence, age, duration of time living with fistula; data related to patient physical screening and clinical diagnosis (incontinence type and surgical diagnoses); fistula cause (obstetric, iatrogenic, traumatic); procedure type and surgeon-rated level of difficulty (simple, intermediate, difficult); and outcome at hospital discharge (fistula closed and dry, fistula closed but incontinent, fistula not closed and incontinent). Data from follow-up visits—for example, due to a change in continence status or a need for additional treatment—was also tracked.

Outreach data captured patient characteristics to assist with tracking and follow-up with clients. Outreach teams also recorded patients' referral method (community outreach worker, health facility, radio, rally, sports, other), outcome of verbal screening at community level using the Obstetric Fistula Community Based Assessment Tool (OF-COMBAT) tentative diagnosis of VVF, urinary incontinence, RVF, 3rd and 4th degree perineal tears, or perineal tears and stool incontinence combined with level of confidence in tentative diagnosis: highly likely, likely, and least likely, and date of referral to hospital [34]. In Phase 2 of the program (May 2017-April 2020), reintegration data capture was added to the standard follow-up procedure. Baseline data were collected at 2-4 weeks post-discharge with follow-up data collected either in-person or over the phone at 3, 6, and 12 months following discharge. The post-repair questionnaire included questions related to continence status (always, sometimes, never), socializing ability (fully, somewhat, not at all), ability to work (fully, somewhat, not at all), functional status (fully back to normal, somewhat back to normal, not at all back to normal), self-esteem (range 1-7), and average monthly household income (<5,000 Kenyan Shillings (KSh), 5,000-10,000 KSh, 10,000-20,000 KSh, 20,000-30,000 KSh, or >30,000 KSh).

### Analysis

Analysis of patient data was descriptive in nature. We characterized the sociodemographic and clinical characteristics of FTN-supported surgeries and patients supported for surgery using proportions, means and standard deviations, and medians and interquartile ranges, per variable type. We then described the surgical outcomes achieved overall as well as the surgical outcomes achieved by surgical procedure type, provider-reported surgical difficulty, incontinence duration, and number of fistula surgeries. Duration of fistula in years was compared across program years using one-way analysis of variance. Participant endorsement of post-repair physical and psychosocial status was described across follow-up data collection periods, with trends over time modeled using generalized least squares regression with random effects to accommodate our longitudinal data structure.

## Results

### Launching the Fistula Treatment Network

**Fig. 3:**
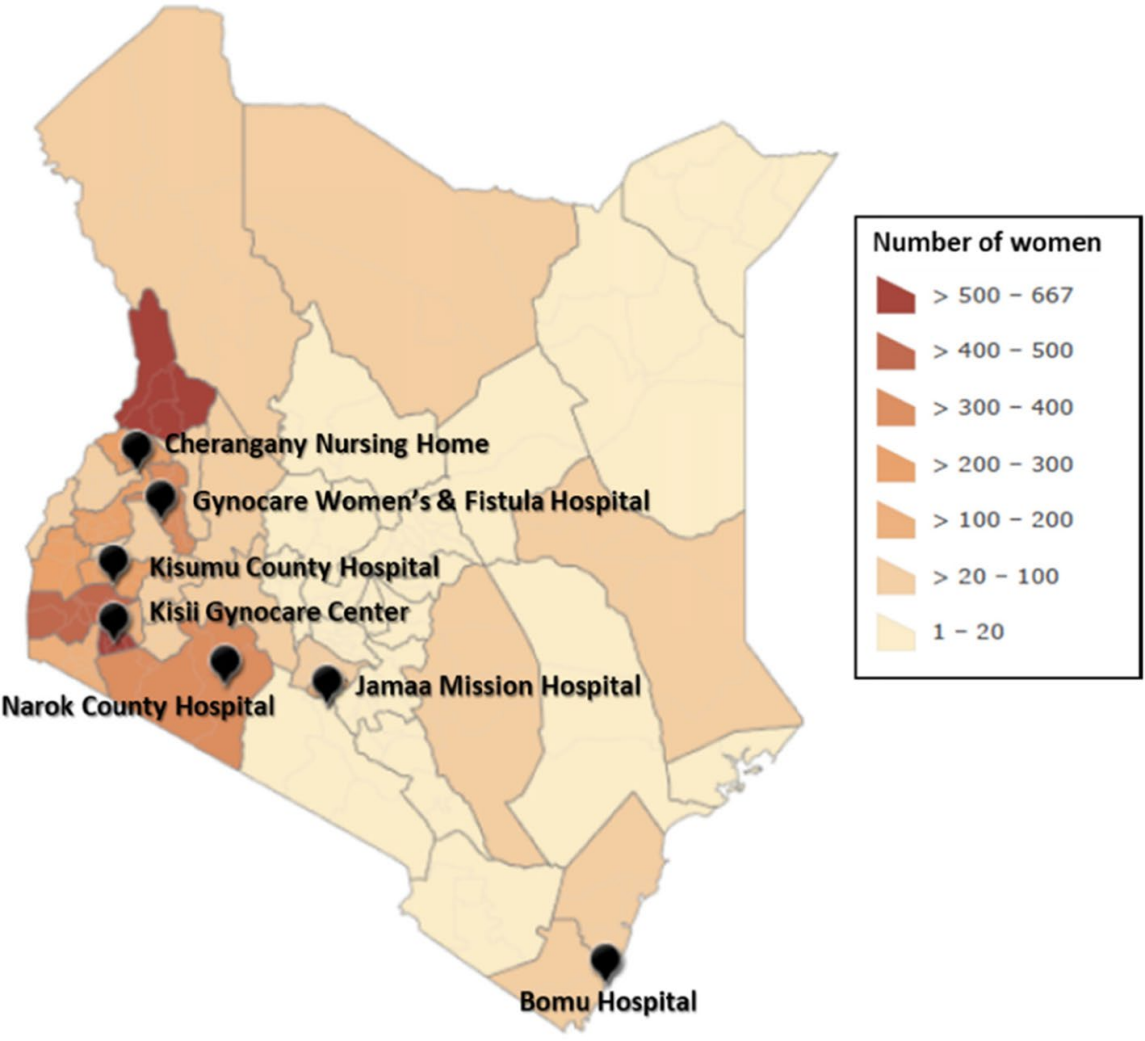
Fistula Treatment Network supported fistula surgery sites and number of women who underwent surgery by county, Kenya, May 2014-April 2020

In 2014, Fistula Foundation launched FTN starting with four hospitals and three outreach organizations. Over the six-year period, FTN grew to seven hospitals and five outreach organizations (Table 1). As seen in Fig. 3, the hospitals were clustered in Nairobi and the western, rift valley, and coastal regions, which closely matches the population density of the country. The outreach locations focused on regions where there was a perceived high burden of fistula. Some of these interventions were based in the county where a treatment center was located, but they also extended far into more rural counties. Women identified in a region where no fistula surgery capacity existed would be referred to the nearest treatment center in a neighboring county. While the community outreach activities undertaken were mostly concentrated in 19 counties, information disseminated through radio and mass media crossed county borders, effectively reaching a much larger audience. Women from all 47 counties of Kenya were referred for treatment through FTN partner hospitals, underscoring the extensive reach of the awareness-raising activities.

**Table 1:** Fistula Treatment Network Partners and Operational Areas May 2014-April 2020

**Fistula Surgery**	**Hospital Location**
Gynocare Women’s and Fistula Hospital	Uasin Gishu (Eldoret)
Cherangany Nursing Home	Trans-Nzoia (Kitale)
Kisumu County Hospital	Kisumu
Kisii Gynocare Center	Kisii
Jamaa Mission Hospital	Nairobi
Narok County Hospital	Narok
Bomu Hospital	Mombasa
**Community Outreach and Reintegration**	**Counties of Coverage**
Women and Development Against Distress in Africa (WADADIA)	Bungoma, West Pokot, Kakamega, Siaya, Baringo, Elgeyo-Marakwet
Daraja Mbili Vision Volunteers (DM)	Kisii, Nyamira, Narok
Maisha Empowerment Initiative (MEI)	Kisumu, Siaya, Homa Bay
Women Education and Health for Development (WOHED)	Garissa, Tana River
Bomu Hospital	Mombasa, Kilifi, Kwale, Taita-Taveta

As the program matured, FTN was able to evaluate the need for fistula care in other regions, and then purposefully establish partnerships to increase surgical capacity alongside community outreach and reintegration services.

### Expand Availability of Routine Fistula Surgery

The Foundation provided payment to FTN treatment sites to cover the full cost of fistula surgery—including screening, pre-and post-operative care, physical therapy, psychosocial counseling, and follow-up care for women who experienced complications or required additional management. The Foundation also provided operating theater equipment, surgical instruments, headlamps, and consumable supplies for the ward, as well as equipment necessary for data collection and documentation (like laptops and cameras). FTN sites also distributed Fistula Care Packs, individual kits of personal hygiene products, to each woman at the time of discharge.

During the program period, a total of 6,223 surgeries were conducted at the seven hospital locations (Table 2). The median patient age at first surgery was 34 (IQR 25-54). Women came from all counties of Kenya, with most patients coming from the western counties of Bungoma (13%), West Pokot (12%), and Kisii (10%; Table 2), as well as from Uganda.

**Table 2:** Characteristics of Fistula Treatment Network supported fistula-related surgeries, Kenya, May 2014-April 2020

	Surgeries		Women	
	n=6,223		n=5,720	
	n	%	n	%
Number of surgeries recorded				
1			5,327	93.1
2			319	5.6
3 or more^a^			74	1.7
Procedure type				
VVF Repair	2,469	39.7	2,251	39.4
3rd/4th Degree Perineal Repair	1,792	28.8	1,718	30.0
RVF Repair	1,350	21.7	1,233	21.6
Urethral Fistula Repair	217	3.5	194	3.4
VVF/RVF Repair	131	2.1	123	2.2
Other^b^	264	4.2	201	3.5
Age, median (IQR)	34 (25-54)		34 (26-45)	
County	(n=6,223)		(n=5,720)	
Bungoma	799	12.8	755	13.2
West Pokot	716	11.5	667	11.7
Kisii	596	9.6	541	9.5
Homa Bay	464	7.5	420	7.3
Uasin Gishu	391	6.3	354	6.2
Narok	353	5.7	328	5.7
Others^c^	2904	46.7	2655	46.4
Incontinence type	(n=6220)		(n=5,717)	
Urinary only	3,045	49.0	2,750	48.1
Fecal only	2,947	47.4	2,756	48.2
Both urinary and fecal	228	3.7	211	3.7
Incontinence duration, in years	(n=6,157)		(n=5,648)	
1 year or less	1,708	27.7	1,556	27.5
1-3 years	974	15.8	889	15.7
3-5 years	661	10.7	605	10.7
5-10 years	995	16.2	921	16.3
10 years or more	1,819	29.5	1,683	29.8
Presumed cause of fistula	(n=6,150)		(n=5,648)	
Obstetric	5,853	95.2	5,400	95.6
Iatrogenic	153	2.5	134	2.4
Traumatic	144	2.3	114	2.0

Notes: ^a^Number of surgeries: 3 (n=51), 4 (n=14), 5 (n=6), 6 (n=2), and 7 (n=1). ^b^Surgery type: ureteric fistula repair (78 surgeries, 70 women), sling or urethroplasty (51 surgeries, 39 women), urinary diversion (51 surgeries, 42 women), vaginoplasty (38 surgeries, 24 women), other (51 surgeries, 40 women). ^c^Other counties (Fig. 3; full specification of counties in Table S1)

The mean time living with fistula-related incontinence was 8.3 years (SD 9.2; not shown); however, the mean number of years living with fistula decreased significantly across program years, with mean 9.3 years (SD 9.9) in 2014-15 compared with 8.1 (SD 9.2) in 2018-2019 (p<0.001).

The majority of women had one surgery recorded in FTN records (93.1%). The most common type of surgical procedure conducted was vesico-vaginal fistula (VVF) repair (39.4%) with repair of 3rd- and 4th-degree perineal tears being the second most common (30.0%; Table 2). The symptoms of unrepaired 3rd- and 4th-degree perineal tears are nearly identical to fistula—incontinence of flatus and stool—and have been fairly commonly observed in other fistula treatment programs in Kenya [35]. Recto-vaginal fistula (RVF) repair comprised 21.6% of cases. The majority of procedures (90.5%) resulted in an outcome of fistula closed and the woman continent at discharge (Table 3). Some variation in surgical outcome was observed by procedure type, level of difficulty, and number of procedures done.

**Table 3:** Fistula Treatment Network supported fistula-related surgeries by outcome, Kenya, May 2014-April 2020

Surgical Characteristics	N	Surgical Outcome					
		Fistula not closed		Fistula closed, incontinent		Fistula closed and continent	
		n	%	n	%	n	%
All procedures^a^	6198	388	6.3	203	3.2	5607	90.5
Procedure type							
VVF repair	2459	233	9.5	129	5.3	2097	85.3
RVF repair	1341	46	3.4	37	2.8	1258	93.8
VVF/RVF repair	130	11	8.5	7	5.4	112	86.2
3rd/4th degree perineal tear repair	1790	45	2.5	15	0.8	1730	96.7
Urinary diversion	46	4	8.7	1	2.2	41	89.1
Vaginoplasty	38	6	15.8	0	0.0	32	84.2
Urethral fistula repair	217	21	9.7	7	3.2	189	87.1
Ureteric fistula repair	78	11	14.1	1	1.3	66	84.6
Sling or urethroplasty	51	5	9.8	5	9.8	41	80.4
Other	48	6	12.5	1	2.1	41	85.4
Procedure difficulty							
Simple	2594	45	1.7	32	1.2	2517	97.0
Intermediate	2338	115	4.9	69	3.0	2154	92.1
Difficult	1257	227	18.1	102	8.1	928	73.8
Incontinence duration, in years							
1 year or less	1701	111	6.5	57	3.4	1533	90.1
1-3 years	971	70	7.2	29	3.0	872	89.8
3-5 years	660	35	5.3	17	2.6	608	92.1
5-10 years	991	54	5.5	31	3.1	906	91.4
10 years or more	1810	115	6.4	65	3.6	1630	90.1
Procedure number							
1	5700	313	5.5	176	3.1	5211	91.4
2	389	49	12.6	21	5.4	319	82.0
3 or more^b^	73	16	21.9	4	5.5	53	72.6

Notes: ^a^ Outcome data were missing for 25 surgeries ^b^ Surgical outcome of fistula closed and continent was achieved by 72.3% of 3rd procedures, 78.3% of 4th procedures, and 46.2% of 5th or higher procedures

### Health provider training and capacity building

The first cohort of two fistula surgeons was trained for a six-week period in September 2014 at the FIGO-accredited Gynocare training center in Eldoret. This initial training was followed by ongoing mentorship of surgeons through surgical workshops, on-site visitation by members of FTN, participation in other surgical training opportunities through partner organizations, and opportunities for exposure to other trainers in different environments. Furthermore, interested doctors with little fistula surgery experience could shadow the trained surgeons prior to full enrollment in the training program, which helped both the doctor and FTN assess if fistula surgery training would be a good fit.

Over the six-year period, FTN supported training for 11 fistula surgeons, seven from Kenya and four from east and southern Africa. The seven Kenyan surgeons trained through FTN conducted 2,003 surgeries during the period. The remaining surgeries (4,220) were conducted by the senior surgeons in FTN who were trained prior to 2014. Four of these surgeons became trainers for FTN and assisted the trainees with ongoing mentorship and guidance. The success rate for the surgeons trained (fistula closed and dry) was 96%. Approximately 58% of the procedures performed by surgeon trainees were 3rd- and 4th-degree tears and RVF repairs, which typically result in higher rates of success.

FTN expanded in 2017 to include more health providers involved in the care and treatment of women with fistula. This expansion was made following feedback from the surgeons at the 2016 Stakeholder Meeting. From 2017 to 2020, 19 nurses from five centers were trained through FTN. Nurse training began with a two-week training at the Gynocare FIGO Training center for two nurses at a time and continued with on-site mentorship visits to the individual centers for ongoing coaching and support. Health providers' participation in FTN also expanded to include surgeons from different disciplines, including urology, plastic surgery, and orthopedics. These providers were valuable in helping to address the needs of the most complex cases. These providers worked together with the network of fistula surgeons, who were primarily gynecologists, to share expertise and find the best solutions for individual patients.

Beyond the clinical training, FTN helped foster a strong community of practice for providers involved in fistula care. Across the network, standardized care protocols were adopted, including clinical management tools, procedures and protocols to improve the quality of patient care and guide decision making, and standardized patient data collection forms. A WhatsApp group was created as a virtual forum to support real-time collaboration and communication between the surgeons. Through this channel surgeons discussed and solicited feedback on treatment plans for complicated cases, and shared information on what worked and what failed, helping other surgeons in the network benefit from the experience of their colleagues.

Annual FTN stakeholder meetings brought together the surgical team and community outreach teams to promote dialog, improve coordination and mutual understanding, and find the best solutions to meet the holistic needs of patients at the hospital and community levels. The surgeons were supported to attend conferences hosted by the International Society of Obstetric Fistula Surgeons (ISOFS) between 2014 and 2019 in Uganda, Nigeria, and Nepal, to learn from colleague surgeons, share their research, and further build a global community of fistula care providers.

### Community Mobilization and Outreach

**Fig. 4:**
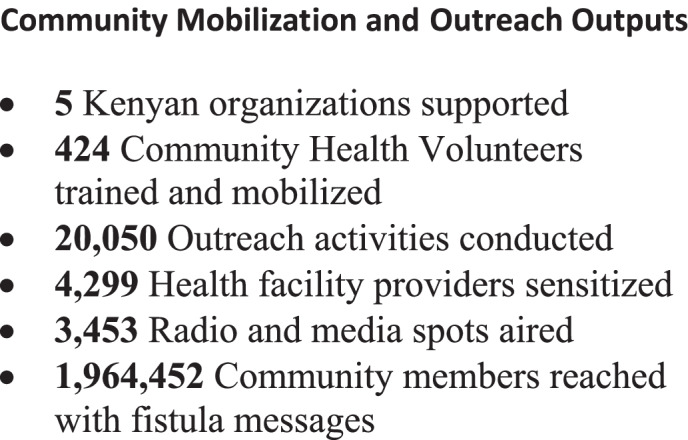
Community Mobilization and Outreach Outputs

FTN implemented a broad range of outreach and mobilization activities (Fig. 4). Key community outreach activities included:

#### Training for Community Health Volunteers (CHVs), Health Providers, and Local Leaders

 CHVs were trained using a curriculum developed by FTN that included modules on fistula prevention, fistula signs and symptoms, screening, identification, referral of clients, post-operative reintegration support, and strategies for effective community engagement. After training, CHVs were given a small monthly stipend and mobile phone airtime allowance to improve communication with communities and patients. CHVs were responsible for facilitating transport for women being referred to the nearest treatment center by providing funding or reimbursement using mobile money (M-pesa). CHVs submitted monthly reporting on the number of women screened and referred for fistula, the number of outreach activities conducted, and the number of community members reached through their efforts. In addition to training CHVs, FTN also trained nurses, midwives, clinical officers, and other cadres of health providers based in health centers in order to improve early diagnosis and referral of women suspected with fistula. Finally, FTN sensitized influential community leaders and chiefs, the gatekeepers in many communities, to support women with fistula and dispel entrenched myths and misperceptions that spread stigma.

#### Community Engagement and Mobilization Activities

FTN partners conducted community meetings and hosted events including rallies and roadshows—which often involved music, community theater, and puppetry—using “info-tainment” to generate excitement and interest while passing important information about fistula. WADADIA used an innovative approach to spread awareness by sponsoring the *Let’s Kick Fistula out of Africa* women’s soccer team that played in the Kenyan Premier League. The team traveled around the country to participate in matches and used the half time as an opportunity to disseminate information to large crowds.

#### Fistula Hotline and IEC Materials

FTN established a Fistula Hotline where people could call for more information or seek a referral for themselves or a family or community member. The hotline number was distributed through mass media including radio announcements and live radio talk shows that were broadcast in local languages throughout the country. FTN also printed and disseminated information, education, and communication (IEC) materials including posters, banners, flyers, tote bags, keyrings, and calendars with the hotline number. Calendars were particularly effective as people kept them on their walls for many years, displaying the photographs and illustrations as part of their home or office décor and providing a good conversation starter for visitors.

FTN worked to improve the verbal screening process to strengthen case identification and referral. The OF-COMBAT was developed to enhance verbal screening to more accurately identify potential fistula cases eligible for referral from cases that were less likely to be fistula (and more likely to be another type of incontinence) [34]. The outreach organizations conducted 6,714 verbal fistula screenings, and 4,690 fistula surgeries were performed for women referred through community outreach activities, representing 75% of total surgeries conducted through the network. Community outreach methods resulting in the most referrals were CHVs, radio messages, and health facility staff sensitization, which were responsible for 63%, 13%, and 10% of referrals, respectively.

To strengthen adoption of community engagement strategies for obstetric fistula, FTN worked with the MOH to develop a training curriculum for CHVs on identification, referral, and support for women living with obstetric fistula. At the time of this writing, FTN was planning a Training of Trainers program for seven county-level facilitators to roll out the curriculum to high-need areas. Additionally, FTN supported other national efforts on obstetric fistula by contributing to the MOH National Strategic Framework to End Female Genital Fistula in Kenya together with AMREF and UNFPA, and by supporting the Beyond Zero Campaign of Kenyan First Lady Hon. Margaret Kenyatta to roll out effective community outreach and reintegration programs in high-need areas.

### Reintegration Post-Repair

**Fig. 5:**
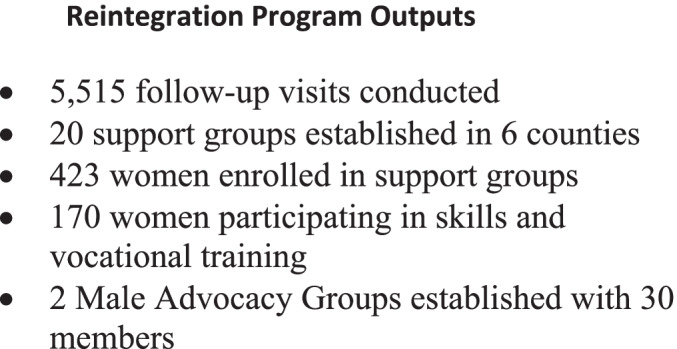
Reintegration Program Outputs

Post-repair reintegration activities included follow-up and monitoring of women after surgery in their home environment, enrollment in survivor-led support groups, income-generating opportunities, vocational training, educational support, and establishment of male advocacy groups (Fig. 5). FTN expanded to invest more heavily in reintegration programming following stakeholder feedback in 2017, the results of which are presented here.

From 2017 to 2020, 2,691 women underwent fistula surgery and were thus eligible for reintegration support. The outreach teams and CHVs conducted follow-up visits, in person or over the phone, to monitor and assess the survivors’ specific needs and determine the appropriate reintegration interventions. During the follow-up visits a questionnaire was administered to assess physical and psychosocial wellbeing. Most participants were followed up with at least once (83.4%), and over half were followed up with at least twice (61.7%) (not shown).

**Fig. 6:**
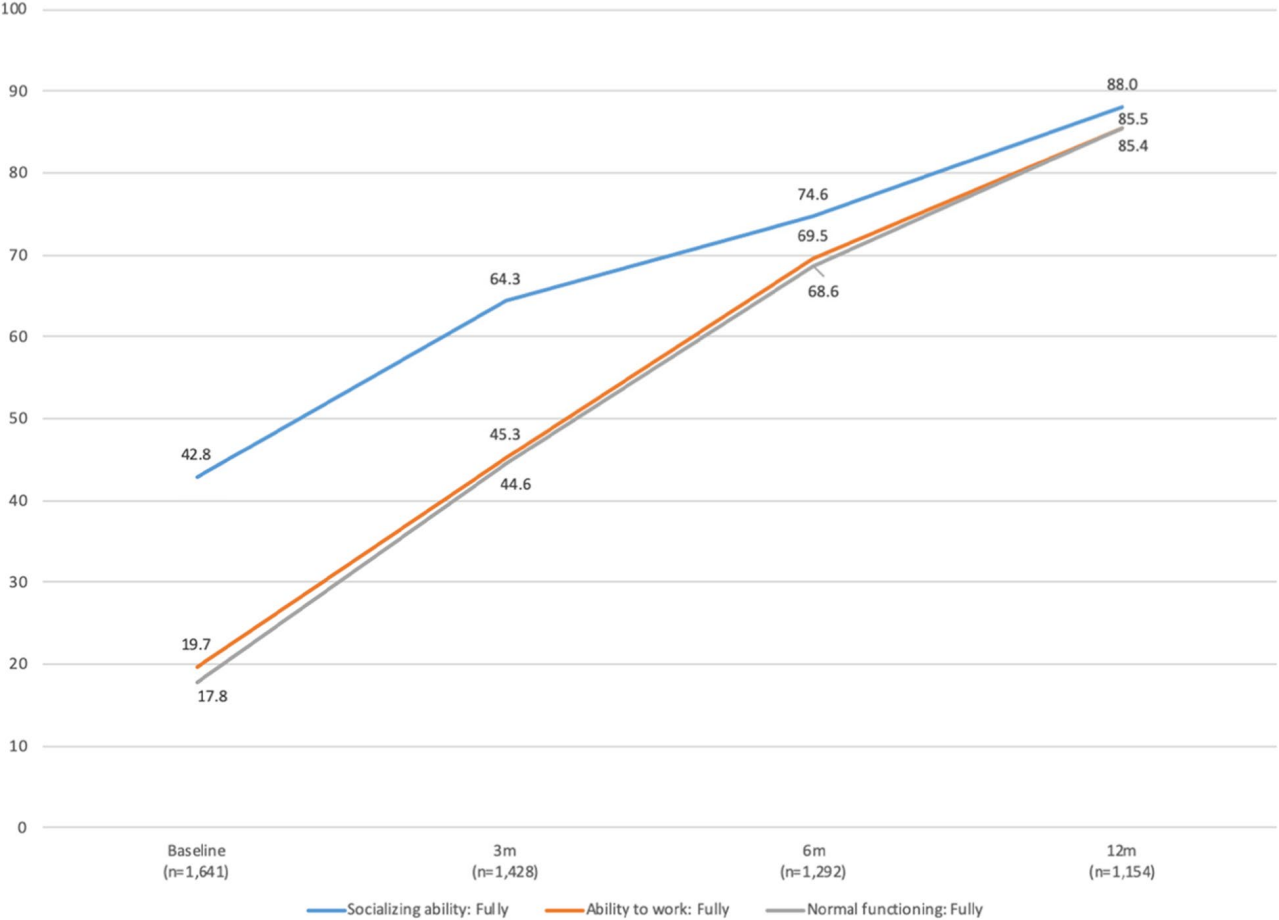
Trajectories of psychosocial status during post-surgical follow-up

The physical and psychological outcomes across the 12 months post repair were encouraging. At 12 months post repair, 96% of women (n=1,092) reported that they were dry and not experiencing any incontinence (Table S2). Increases in socializing, working ability, and normal functioning from baseline were substantial through 12 months (Fig. 6). Over the 12-month follow-up period, the proportion of women reporting being able to socialize “fully” with family and friends increased from 43 to 86%, the proportion of women reporting being able to fully work increased from 20 to 86%, and the proportion of women reporting normal functioning increased from 18 to 85%. Mean self-esteem increased from 4.3 at baseline (SD 1.5) to 6.3 (SD 1.4) at 12 months (Table S2).

Twenty support groups in six counties of Kenya were established and 423 women became participating members. The support groups established their own structures and elected their own officers to govern meetings. The support groups were comprised of between 20 and 25 members each, and typically met monthly. Nearly half (9) went on to register as Self-Help CBOs with the Kenyan government, which allows them access to greater opportunities and resources.

Beyond emotional and psychological support, these groups were a platform for income generation. The different groups selected the activities they felt best suited their members or the market in which they lived, including farming/horticulture, poultry farming, goat and cattle rearing, fish farming, and catering, and FTN provided seed funding to support these activities. The members also participated in table banking and merry-go-round savings practices, whereby members would all contribute a small amount of money to the kitty each month and would rotate access to those funds to each member in turn. FTN also supported women to enroll in training programs in tailoring, hairdressing, bead making, and computer literacy. Roughly 60% of women who graduated from skills programs have been able to turn the knowledge and skills acquired into practice by starting their own business, and others have been employed by small business owners in their counties. Most of the women utilizing their skills have reported improved income and nutrition at their household level. For example, the proportion of participants reporting monthly household income <5,000 KSh reduced from 93.5 to 67.81% over time (not shown). Women have acquired some degree of financial independence, which gives them the ability to make informed choices about their lives.

A powerful part of the reintegration support was seeing that women with lived experience of fistula can be powerful ambassadors to help identify and support other women with fistula from their communities. These fistula survivors are also effective advocates in mobilizing their communities around treatment and prevention messages and health-seeking behaviors. *Voices of Hope and Action: A Fistula Survivors Movement* has started by WADADIA, whose aim is to train and empower women with lived experience of fistula to be able to advocate for improved access to sexual and reproductive health services and to create more visibility and awareness of the particular needs of this population.

Finally, Male Advocacy Groups were created so men could be better informed to talk with other men in their communities about fistula, about the importance of supporting their partners with this condition, and about preventing fistula by encouraging women to go to a health center or hospital to give birth with a skilled birth attendant instead of at home or in the village, as has long been the tradition. Involving the husband and other male family members in the fistula survivor’s treatment also helped improve adherence to post-repair instructions such as abstaining from sexual intercourse during the recovery period.

## Discussion

The Kenya FTN sought to increase access to treatment for women living with fistula and develop the capacity of health providers, hospitals, and CBOs to strengthen fistula care and reintegration support. Results from process and outcome assessment highlight program achievements and the positive impact of fistula repair surgery and follow-up on women’s continence status and quality of life. The data shows clear improvement in measures of social and emotional health and livelihood across the 12 months women were followed up with after fistula surgery.

The Kenya FTN highlighted that community outreach is fundamental to case identification and referral of patients. Women with fistula are highly stigmatized, isolated, and often live in hard-to-reach rural areas [4–6]. 75% of women who were treated through FTN came as a direct result of CBO engagement, the majority of which occurred through CHVs who developed trust and confidence with their community members and could provide ongoing support over a prolonged period. The creation of the OF-COMBAT screening tool also helped improve the identification and referral of patients and helped community members explain to women how a referral decision was reached. This helped build confidence in the program and reduced program transportation costs by ensuring only women who likely had fistula were referred to the treatment center.

While FTN sought to expand access to care by increasing the number of treatment sites and trained surgeons providing fistula care, it was also essential to ensure the surgical services were high quality. FTN strategically decided to provide training and ongoing mentorship to a smaller number of surgeons as opposed to spreading training over a large number of surgeons in every county. Adequately training a fistula surgeon requires sufficient clinical exposure during their training, a large enough case load in their respective home institution to maintain and improve their surgical skills, and a referral mechanism for cases outside their competency level. In the second phase of FTN, after the treatment network was largely established, the network focused training activities almost entirely on building capacity of those already trained to take on more complex cases. The 2021 UNFPA Obstetric Fistula Manual suggests that this approach, of “quality over quantity” when it comes to training specialist fistula surgeons, is the best way to ensure that women have access to truly expert care and optimizes the likelihood of a successful surgical outcome [8]. This strategic approach required strong case identification and referral systems linking communities to specialists at limited target facilities—both of which were critical components of the FTN approach.

Still, finding and retaining fistula surgeons to be based in geographically strategic locations was a challenge. During this project period, FTN was unable to find a resident surgeon from coastal Kenya motivated to train in fistula surgery. Instead, FTN opted to fly out a Nairobi-based fistula surgeon every quarter to provide surgery, which allowed for consistency, on-site mentorship of the resident team, and improved follow-up care for women who required multiple interventions. Additionally, two of the seven surgeons trained through FTN opted out of the program following completion of their training, despite sustained efforts by FTN to motivate and incentivize this work.

Post-repair reintegration activities across FTN were strengthened following stakeholder feedback that this was an essential missing link to support more holistic healing and empowerment for women with fistula. Research has shown that this component of fistula care improves physical, psychosocial, and economic outcomes for women, and is supported by many fistula stakeholders, [30] but is often overlooked and under-resourced.

FTN reintegration activities also provided an opportunity for community teams to collect data on women’s long-term physical and psychosocial outcomes that was not obtainable through the treatment center, providing greater insight into the trajectory of post-repair recovery and contributing to the relatively limited literature on long-term continence status and quality-of-life outcomes for women after fistula repair. While appropriate controls are not available to robustly compare the impact of FTN our reintegration programming, and the literature suggests that successful surgery alone is associated with substantial improvements in quality of life, [4, 11, 13, 36] the few published controlled interventions incorporating psychosocial support report significantly improved self-esteem and reduced suicidal ideation, with non-significant intervention differences in depression, anxiety, and post-traumatic stress disorder. However, these studies provided only short-term psychosocial support that did not extend past hospital discharge. Other uncontrolled psychosocial support interventions programs have reported increased confidence, self-esteem, and emotional health [37–39]. Our findings on improving socializing ability, ability to work, and normal functioning in the post-repair period follow a similar pattern to the increases in reintegration reported over the 12 months following surgery by El Ayadi et al. in Uganda [12]. However, compared with El Ayadi et al., we observed somewhat greater increases over time, including after six months following surgery, after which El Ayadi et al. observed less steep gains. These discrepancies may be explained by the higher surgical success rates seen in our study and the ongoing interventions received by our program participants. While the program components that FTN implemented are supported theoretically, there is a need for more robust research designs that would enable us to test comparative effectiveness of these components, and inform the cost effectiveness of various approaches. FTN has since adapted its data collection instruments to allow for better understanding of outcomes across levels of support group participation and types of economic empowerment interventions.

With the large number of community and hospital partners across FTN, data collection and management was one of the more challenging aspects of the program. Routine monitoring of facility and community data by Fistula Foundation staff helped improve completion and accuracy of reports from FTN partners but this process required continuous oversight. Data collection tools were limited to paper records and Microsoft Excel; use of a relational database or mobile device data collection tools may have helped improve the ability of FTN to manage large amounts of program data. Ultimately, we would also want fistula data to be included in the Kenya health management information system (HMIS) II System so that the data on fistula prevalence is incorporated into national data sets, as visibility on the issue remains low in part due to limited data availability.

FTN’s patient-centric approach placed high value on delivering high-quality, comprehensive, and timely fistula treatment. This priority resulted in FTN establishing partnerships with both private and public hospitals, wherever it could assure regular availability of high-quality surgery and post-operative care. Challenges within the public hospitals—lack of operating theater availability, limited ward space, human resource constraints, and shortage of essential supplies and equipment for fistula care—could be disruptive to fistula services. As a result, between 2014 and 2020, the majority of hospitals in FTN (five of seven) were private facilities. Since 2020, FTN has worked more closely with the MOH, and a greater emphasis has been made to integrate public facilities into FTN. In 2021, the program added two County Referral Hospitals (Vihiga and Garissa County) and expanded outreach coverage to those areas through the county health structures. Surgeons and nurses from the County Hospitals have been identified and trained as part of FTN. The partnership with the MOH to roll out the National Female Genital Fistula Training Curriculum for Community Health Volunteers will ensure that more CHVs across the country will be oriented on fistula, which will help improve long-term capacity for identification, referral, and support for women with fistula.

Thoughtful integration of fistula care and service delivery into the different levels of the health system could help improve long-term sustainability of these services. However, a persistent challenge exists with financing fistula care, a situation not unique to Kenya. In resource-constrained settings, Ministries of Health must make difficult decisions about health care spending, and funding for fistula care is often not given priority. Because women with fistula are often poor and unable to afford the cost of their care, if governments are not able to heavily subsidize or fully cover the cost of fistula care, donor funding will be essential to continue providing these services. The reason Fistula Foundation exists and created FTN was to ensure women with fistula get the holistic care and support they deserve.

The strength of the FTN model is more than its ability to markedly improve access to treatment. It also provides a holistic support system for women with fistula, including community identification, referral to the hospital for fistula surgery, and reintegration. The integration of local CBOs and public and private hospitals created a unique structure that allowed women to get the best care while also allowing each partner to focus on its strengths, with hospitals and surgeons concentrating on clinical care and CBOs focusing on improving awareness, case identification, referral, and reintegration. FTN sought to address each aspect of the treatment journey for women, removing financial, psychosocial, and logistical barriers to expedite their ability to receive the care they required. Additionally, for many women enrolled in the reintegration programs, FTN sought to move them beyond the status quo following surgery and to elevate their overall psychosocial health and economic wellbeing.

FTN network was conceptualized, implemented and expanded using a stakeholder-involved approach to address gaps in the continuum of fistula care within Kenya. Transportability of the model was not a focus of the current project; however, the lessons learned during the FTN implementation process are likely relevant to other settings where fistula is prevalent and where health systems are similar. Future research informed by implementation science frameworks to understand transportability and adaptation considerations for other settings is warranted.

## Conclusion

The Kenya Fistula Treatment Network is an effective model that created linkages across the health system and community to support women with fistula through all aspects of their healing journey to achieve the best possible outcomes. In the first six years of program implementation (2014-2020), FTN improved awareness of fistula and reduced stigma, increased access to fistula care services, strengthened the fistula care workforce, and enhanced post-operative follow-up and reintegration support. FTN continues to operate and expand with financial support from Fistula Foundation and private donors. This integrated approach can be a useful model for delivery of comprehensive services in other countries where the burden of obstetric fistula is high.

## Supplementary Information

**Additional file 1.**

**Additional file 2.**

## Data Availability

The datasets used and/or analyzed during the current study are available from the corresponding author on reasonable request.

## Abbreviations

FTN: Fistula Treatment Network
CBO: Community Based Organization
CHV: Community Health Volunteer
FIGO: International Federation of Gynecology and Obstetrics
IEC: Information, Education and Communication
ISOFS: International Society of Obstetric Fistula Surgeons
MOH: Ministry of Health
NGO: Non-governmental Organization
OF-COMBAT: Obstetric Fistula Community Based Assessment Tool
UNFPA: United Nations Populations Fund
WADADIA: Women and Development against Distress in Africa
