# Supplementary material for: Building a country-wide Fistula Treatment Network in Kenya: results from the first six years (2014-2020)
Source: BMC Health Serv Res. 2022 Mar 1;22:280. doi: 10.1186/s12913-021-07351-x (PMC8889651; doi:10.1186/s12913-021-07351-x)
Supplement: Supplementary file 1 — Additional file 1. [file 12913_2021_7351_MOESM1_ESM.docx]

**SUPPLEMENTAL TABLES**

**Table S1. Patient residence for Fistula Treatment Network supported fistula surgeries, Kenya, May 2014 – April 2020**

| County | Surgeries | | Women | |
| --- | --- | --- | --- | --- |
|  | n=6,223 | | n=5,720 | |
|  | n | % | n | % |
| Baringo | 98 | 1.6 | 82 | 1.4 |
| Bomet | 49 | 0.8 | 46 | 0.8 |
| Bungoma | 799 | 12.8 | 755 | 13.2 |
| Busia | 89 | 1.4 | 79 | 1.38 |
| Elgeyo-Marakwet | 47 | 0.8 | 42 | 0.7 |
| Embu | 2 | 0.0 | 2 | 0.0 |
| Garissa | 54 | 0.9 | 48 | 0.8 |
| Homa Bay | 464 | 7.5 | 420 | 7.3 |
| Isiolo | 5 | 0.1 | 4 | 0.1 |
| Kajiado | 23 | 0.4 | 19 | 0.3 |
| Kakamega | 236 | 3.8 | 214 | 3.7 |
| Kericho | 54 | 0.9 | 48 | 0.8 |
| Kiambu | 25 | 0.4 | 22 | 0.4 |
| Kilifi | 78 | 1.3 | 76 | 1.3 |
| Kisii | 596 | 9.6 | 541 | 9.5 |
| Kirinyaga | 1 | 0.0 | 1 | 0.0 |
| Kisumu | 248 | 4.0 | 230 | 4.0 |
| Kitui | 28 | 0.5 | 28 | 0.5 |
| Kwale | 77 | 1.2 | 76 | 1.3 |
| Laikipia | 8 | 0.1 | 7 | 0.1 |
| Lamu | 9 | 0.1 | 9 | 0.2 |
| Machakos | 11 | 0.2 | 11 | 0.2 |
| Makueni | 11 | 0.2 | 10 | 0.2 |
| Mandera | 20 | 0.3 | 18 | 0.3 |
| Marsabit | 22 | 0.4 | 22 | 0.4 |
| Meru | 25 | 0.4 | 20 | 0.4 |
| Migori | 193 | 3.1 | 169 | 3.0 |
| Mombasa | 145 | 2.3 | 142 | 2.5 |
| Muranga | 11 | 0.2 | 8 | 0.1 |
| Nairobi | 119 | 1.9 | 114 | 2.0 |
| Nakuru | 82 | 1.3 | 68 | 1.2 |
| Nandi | 99 | 1.6 | 92 | 1.6 |
| Narok | 353 | 5.7 | 328 | 5.7 |
| Nyamira | 113 | 1.8 | 105 | 1.8 |
| Nyandura | 6 | 0.1 | 6 | 0.1 |
| Nyeri | 8 | 0.1 | 6 | 0.1 |
| Samburu | 17 | 0.3 | 14 | 0.2 |
| Siaya | 238 | 3.8 | 210 | 3.7 |
| Taita-Taveta | 11 | 0.2 | 10 | 0.2 |
| Tana River | 17 | 0.3 | 14 | 0.2 |
| Tharaka-Nithi | 12 | 0.2 | 12 | 0.2 |
| Trans-Nzoia | 276 | 4.4 | 257 | 4.5 |
| Turkana | 35 | 0.6 | 25 | 0.4 |
| Uasin Gishu | 391 | 6.3 | 354 | 6.2 |
| Vihiga | 28 | 0.5 | 26 | 0.5 |
| Wajir | 10 | 0.2 | 8 | 0.1 |
| West Pokot | 716 | 11.5 | 667 | 11.7 |
| Uganda | 264 | 4.2 | 255 | 4.5 |

**Table S2. Physical and Psychosocial Health Following Surgery by Post-repair Period,**

**Women who underwent fistula surgery in Fistula Treatment Network, Kenya, May 2017– April 2020**

|  | Baseline | | 3 Mo | | 6 Mo | | 12 Mo | | P-value^a^ |
| --- | --- | --- | --- | --- | --- | --- | --- | --- | --- |
|  | n=1,641 | | n=1,428 | | n=1,292 | | n=1,154 | |  |
|  | n | % | n | % | n | % | n | % |  |
| Socializing ability | (n=1,607) | | (n=1,400) | | (n=1,277) | | (n=1,135) | | <0.001 |
| Not at all | 37 | 2.3 | 33 | 2.4 | 13 | 1.0 | 14 | 1.2 |  |
| Somewhat | 883 | 55.0 | 467 | 33.4 | 311 | 24.4 | 122 | 10.8 |  |
| Fully | 687 | 42.8 | 900 | 64.3 | 953 | 74.6 | 999 | 88.0 |  |
| Ability to work | (n=1,610) | | (n=1,400) | | (n=1,274) | | (n=1,137) | | <0.001 |
| Not at all | 44 | 2.7 | 33 | 2.4 | 16 | 1.3 | 15 | 1.3 |  |
| Somewhat | 1,249 | 77.6 | 733 | 52.4 | 372 | 29.2 | 150 | 13.2 |  |
| Fully | 317 | 19.7 | 634 | 45.3 | 886 | 69.5 | 972 | 85.5 |  |
| Normal functioning | (n=1,607) | | (n=1,400) | | (n=1,275) | | (n=1,137) | | <0.001 |
| Not at all | 51 | 3.2 | 34 | 2.4 | 15 | 1.2 | 16 | 1.4 |  |
| Somewhat | 1,270 | 79.0 | 742 | 53.0 | 385 | 30.2 | 150 | 13.2 |  |
| Fully | 286 | 17.8 | 624 | 44.6 | 875 | 68.6 | 971 | 85.4 |  |
| Self-esteem | (n=1,584) | | (n=1,390) | | (n=1,272) | | (n=1,136) | | <0.001 |
| Mean (SE) | 4.30 (1.46) | | 5.04 (1.38) | | 5.83 (1.32) | | 6.29 (1.41) | |  |
| Continence status | (n=1,610) | | (n=1,404) | | (n=1,277) | | (n=1,137) | | <0.001^b^ |
| Always leaking | 59 | 3.7 | 52 | 3.7 | 34 | 2.7 | 20 | 1.8 |  |
| Sometimes leaking | 56 | 3.5 | 41 | 2.9 | 30 | 2.4 | 25 | 2.2 |  |
| Dry | 1,495 | 92.9 | 1,311 | 93.4 | 1,213 | 95.0 | 1,092 | 96.0 |  |

*^a^P-value for time trend in mean response.* ^b^ *Dry versus always* *or sometimes leaking.*
