# Supplementary material for: Building a country-wide Fistula Treatment Network in Kenya: results from the first six years (2014-2020)
Source: BMC Health Serv Res. 2022 Mar 1;22:280. doi: 10.1186/s12913-021-07351-x (PMC8889651; doi:10.1186/s12913-021-07351-x)
Supplement: Supplementary file 2 — Additional file 2. [file 12913_2021_7351_MOESM2_ESM.pdf]

Self-Certification Form: Determining Whether Human Subjects Are Involved in Research When Obtaining Coded Private Information (Data) and/or Biological Specimens

Instructions:

1. Use this form if you need to provide funding agencies, administrators or collaborators with documentation that your research project does not require IRB review at UCSF. Keep a copy of the form in the PI's research file. Do **not** submit a copy to the IRB.

2. For help making this determination, review the Human Subjects Research Decision Tree and the Not Human Subjects Research guidance page. Contact the IRB at 415-476-1814 or IRB@ucsf.edu with questions.

3. Do not use this form for human stem cell research, which requires review by the GESCR Committee and may require IRB review.

**Principal Investigator:**		
Name and Degree: Alison El Ayadi, ScD MPH	Institution: UCSF	Department: Obs/Gyn/RS
Mailing Address: 550 16th Street, San Francisco, CA 94158	Phone Number: 617.877.7424	E-mail Address: alison.elayadi@ucsf.edu

Study/Grant Title/Award No.:

Building a Country-wide Network for Obstetric Fistula Treatment in Kenya

If your research meets the following conditions, the use of de-identified or coded private information (data) and/or biological specimens does not meet the definition of a human subject and does not require IRB review at UCSF:

1. The research is not regulated by the Food and Drug Administration (FDA) **AND**

2. No one on the UCSF research team has access to identifiable information because one or both of the following apply (check all applicable boxes):

- [x] The researcher(s) receive de-identified data or specimens.
- [ ] The researcher(s) receive coded data or specimens AND one or more of the following apply:
  - The key to decipher the code is destroyed before the research begins, OR
  - The PI and holder of the key enter into an agreement prohibiting the release of the key under any circumstances, OR
  - There are IRB-approved written policies for the repository or data management that prohibit the release of the key, OR
  - There are other legal requirements prohibiting the release of the key under any circumstances.

Principal Investigator's Certification:
I certify that the information provided in this application is complete and correct.

Alison M. El Ayadi — 4/18/2021

Principal Investigators Signature — **Date**
